# Predictors of Occurrence and Severity of First Time Low Back Pain Episodes: Findings from a Military Inception Cohort

**DOI:** 10.1371/journal.pone.0030597

**Published:** 2012-02-15

**Authors:** Steven Z. George, John D. Childs, Deydre S. Teyhen, Samuel S. Wu, Alison C. Wright, Jessica L. Dugan, Michael E. Robinson

**Affiliations:** 1 Department of Physical Therapy and Center for Pain Research and Behavioral Health, University of Florida, Gainesville, Florida, United States of America; 2 U.S. Army-Baylor University Doctoral Program in Physical Therapy (MCCS-HGE-PT), Army Medical Department Center and School, Fort Sam Houston, Texas, United States of America; 3 Department of Biostatistics, University of Florida, Gainesville, Florida, United States of America; 4 Department of Clinical and Health Psychology and Center for Pain Research and Behavioral Health, University of Florida, Gainesville, Florida, United States of America; Finnish Institute of Occupational Health, Finland

## Abstract

Primary prevention studies suggest that additional research on identifying risk factors predictive of low back pain (LBP) is necessary before additional interventions can be developed. In the current study we assembled a large military cohort that was initially free of LBP and followed over 2 years. The purposes of this study were to identify baseline variables from demographic, socioeconomic, general health, and psychological domains that were predictive of a) occurrence; b) time; and c) severity for first episode of self-reported LBP. Baseline and outcome measures were collected via web-based surveillance system or phone to capture monthly information over 2 years. The assembled cohort consisted of 1230 Soldiers who provided self-report data with 518 (42.1%) reporting at least one episode of LBP over 2 years. Multivariate logistic regression analysis indicated that gender, active duty status, mental and physical health scores were significant predictors of LBP. Cox regression revealed that the time to first episode of LBP was significantly shorter for Soldiers that were female, active duty, reported previous injury, and had increased BMI. Multivariate linear regression analysis investigated severity of the first episode by identifying baseline predictors of pain intensity, disability, and psychological distress. Education level and physical fitness were consistent predictors of pain intensity, while gender, smoking status, and previous injury status were predictors of disability. Gender, smoking status, physical health scores, and beliefs of back pain were consistent predictors of psychological distress. These results provide additional data to confirm the multi-factorial nature of LBP and suggest future preventative interventions focus on multi-modal approaches that target modifiable risk factors specific to the population of interest.

## Introduction

In general populations low back pain (LBP) is the most prevalent form of chronic musculoskeletal pain [1] often leading to disability [2], [3]. In military populations musculoskeletal pain has an adverse effect by frequently causing medical evacuation [4] and LBP in particular a common reason for long term disability [5]. As a result of its negative impact prevention of LBP has remained a research priority for both general [6] and military populations [4], [7].

Factors involved in the transition from acute to chronic LBP have been a recent focus of disability prevention research. Such an approach is consistent with secondary prevention [8], and studies in this area have provided important information on effective management of acute LBP. Secondary prevention studies have highlighted psychological influence on the development of chronic LBP [9] and identified patient subgroups that have larger treatment effects when matched treatment is applied [10], [11]. The focus on secondary prevention has been productive in reducing disability from acute episodes of LBP, but there remains the potential of primary prevention for limiting the negative impact of LBP.

The goal in primary prevention is to reduce the overall number of LBP episodes experienced by a population [12]. In contrast to secondary prevention, primary prevention attempts to reduce those that transition from a pain free state to one of experiencing LBP. Back schools, lumbar supports, and ergonomic interventions have all been studied for primary prevention of LBP, but with limited success [13], [14], [15]. These primary prevention studies suggest that more work needs to be completed in determining what factors are predictive of developing LBP before additional preventative interventions can be developed. For example, if modifiable factors are identified as being predictive of LBP then they may provide logical treatment targets for future LBP prevention trials [6].

The purpose of this paper was to report predictors of first time LBP episodes self-reported during 2 years of military duty. This purpose is consistent with primary prevention priorities highlighted in the literature [6], [14]. To best study the development of LBP it is necessary to recruit a group of healthy subjects and follow these subjects until some develop LBP. Furthermore, development of LBP is believed to be multi-factorial in nature so consideration of a range of potential predictors is warranted. In the current study we assembled a large military cohort that was initially free of LBP and included potential predictors from demographic, socioeconomic, general health, and psychological domains. The primary purposes of this study were to identify variables from these domains that were predictive of a) occurrence and b) time to first episode of LBP. Our secondary purposes were to identify variables that were predictive of a) higher pain intensity; b) disability; or c) psychological distress during the first LBP episode.

## Methods

### Ethics Statement

The institutional review boards at the Brooke Army Medical Center (Fort Sam Houston, Texas) and the University of Florida (Gainesville, FL) granted ethical approval for this project. All subjects provided written informed consent prior to their participation.

### Overview

This study was part of the Prevention of Low Back Pain in the Military (POLM) cluster randomized trial [16]. The POLM trial has been registered at ClinicalTrials.gov (http://clinicaltrials.gov) under NCT00373009.

The primary aim of the POLM trial was to determine if core-stabilization exercise and psychosocial education resulted in decreased LBP incidence during 2 years of military duty. POLM trial results indicated that psychosocial education was found to be preventative of seeking healthcare for LBP [17]. This study reports on a secondary aim of the POLM trial which was to determine what factors were predictive of self-reported LBP for Soldiers that responded to a web-based or phone survey tools.

### Subjects

Consecutive subjects entering a training program at Fort Sam Houston, TX to become a combat medic in the U.S. Army were considered for participation from February 2007 to March 2008. Research staff at Fort Sam Houston, Texas introduced the study to individual companies of Soldiers and screened potentially eligible Soldiers.

Subjects were required to be 18–35 years of age (or 17 year old emancipated minor), participating in training to become a combat medic, and be able to speak and read English. Subjects with a prior history of LBP were excluded. In this study a prior history of LBP was operationally defined as a previous episode of LBP that limited work or physical activity, lasted longer than 48 hours, and caused the subject to seek health care. Subjects were also excluded if they were currently seeking medical care for LBP; unable to participate in unit exercise due to other musculoskeletal injury; had a history of lower extremity fracture (stress or traumatic); were pregnant; or if they had transferred from another training group. Other possible exclusions included Soldiers who were being accelerated into a company already randomized or Soldiers who were being re-assigned to a different occupational specialty.

### Exercise and Education Programs

Companies of Soldiers were randomly assigned to exercise and/or education programs as part of the cluster randomized trial [16]. The assigned exercise and education programs are not a focus of this current paper, but are briefly reviewed as we included these as predictive variables in our statistical analyses. All exercise programs were performed in a group setting under the direct supervision of their drill instructors as part of daily unit physical training. The traditional exercise program (TEP) was selected from commonly performed exercises that target the rectus abdominus and oblique abdominal muscles. The core stabilization exercise program (CSEP) was selected from exercises that target deeper trunk muscles that attach to the spine; such as the transversus abdominus, multifidus, and the erector spinae. The TEP and CSEP are described in more detail in previous POLM publications [18], [19].

The brief psychosocial education program (PSEP) involved attendance at 1 session during the first week of training. The session involved an interactive lecture led by study personnel (ACW, JLD) lasting approximately 45 minutes. The lecture consisted of a visual presentation followed by a question and answer session. The PSEP provided Soldiers current, evidence based information on LBP such as stressing that anatomical causes of LBP are not likely and encouraging active coping in response to LBP. Educational material was also provided to the Soldiers by issuing each *The Back Book* as has been done in other LBP trials [20], [21], [22]. The PSEP is described in more detail in a previous publication [23].

### Measures

Baseline measures were collected under supervision of study personnel. Outcome measures were collected via web-based surveillance system or phone to capture monthly information over 2 years.

#### Baseline measures

Soldiers completed standard questionnaires to assess variables consistent with demographic and socioeconomic domains. The information collected included such variables as age, sex, education, income level, smoking history, previous activity level, previous injury, physical fitness scores, and military status. Soldiers also completed self-report measures to assess general health and psychological domains. The Medical Outcomes Survey 12-Item Short-Form Health Survey was used as a self-report of health status for physical (SF-12 PCS) and mental function (SF-12 MCS) [24]. The Back Beliefs Questionnaire (BBQ) was used to quantify beliefs about LBP related to management and outcome [25]. The State-Trait Anxiety Questionnaire (STAI) [26] and Beck Depression Inventory (BDI) [27], [28], [29] were used to measure negative affect from generalized anxiety and generalized depression, respectively. Finally, 9 items from the Fear of Pain Questionnaire (FPQ-III) were used to measure fear about specific situations that normally produce pain [30], [31], [32].

#### Outcome measures

Soldiers were trained in a computer lab on how to use the POLM web-based outcome surveillance system and all assessments were provided through a secure web-site that protected Soldier confidentiality. Access to the system was prompted by an email which was sent to the Soldier's official military email address on the 1st of each month. Additional emails were sent on the 3rd of the month, and again on the 7th of the month if the Soldier still had not responded. Soldiers were queried whether they had experienced any LBP in the last calendar month by email, and this information was used to determine the initial episode of LBP after completing training. Soldiers not responsive to multiple email requests were contacted by phone at the end of 12 and 24 months to determine if LBP had occurred in the past year. Those Soldiers responding to the phone interview were included with the email survey results because the structure of the phone interview was parallel to the email survey. Soldiers successfully contacted by phone completed the same information as they would have if using the web-based system. These self reported incidence data were used as outcomes of interest for our primary purpose – determining baseline predictors for the occurrence and time to first LBP episode.

Soldiers reporting any LBP answered additional questionnaires so that the severity of the first episode could be determined. These measures included pain intensity with a numerical 0–10 rating scale (NRS) [33], disability with the Oswestry Disability Questionnaire (ODQ) [34], [35], physical activity and work fear-avoidance beliefs with the Fear-Avoidance Beliefs Questionnaire (FABQ-PA and FABQ-W) [36], and pain catastrophizing by the Pain Catastrophizing Scale (CAT) [37]. Soldiers were also asked to report days of limited military duty in the past 30 days associated with the initial episode of LBP. These data were used as outcomes of interest for our secondary purpose – determining baseline predictors for the severity of LBP episodes.

### Data analysis

All statistical analyses were performed using the SAS software, version 9 (SAS Institute Inc, 1996) with a type I error rate of 0.05. All authors had full access to all of the data reported in the study and can take responsibility for data integrity and accuracy. Descriptive data for the baseline variables were computed. Then multivariate logistic regression analysis was used to determine baseline predictors of reporting the first episode of LBP and multivariate Cox regression analysis was used to determine baseline predictors of time to reporting first episode of LBP. In the severity analyses, multivariate linear regression was used to determine baseline predictors of pain intensity, disability, and psychological distress. In the regression analyses baseline predictors were considered from all the individual variables from the demographic, socioeconomic, general health, and psychological domains. All variables were entered simultaneously into the regression models to determine which were predictive of the outcome of interest while controlling for other potential variables. Therefore, only adjusted estimates are reported in the results.

## Results

**Figure 1:**
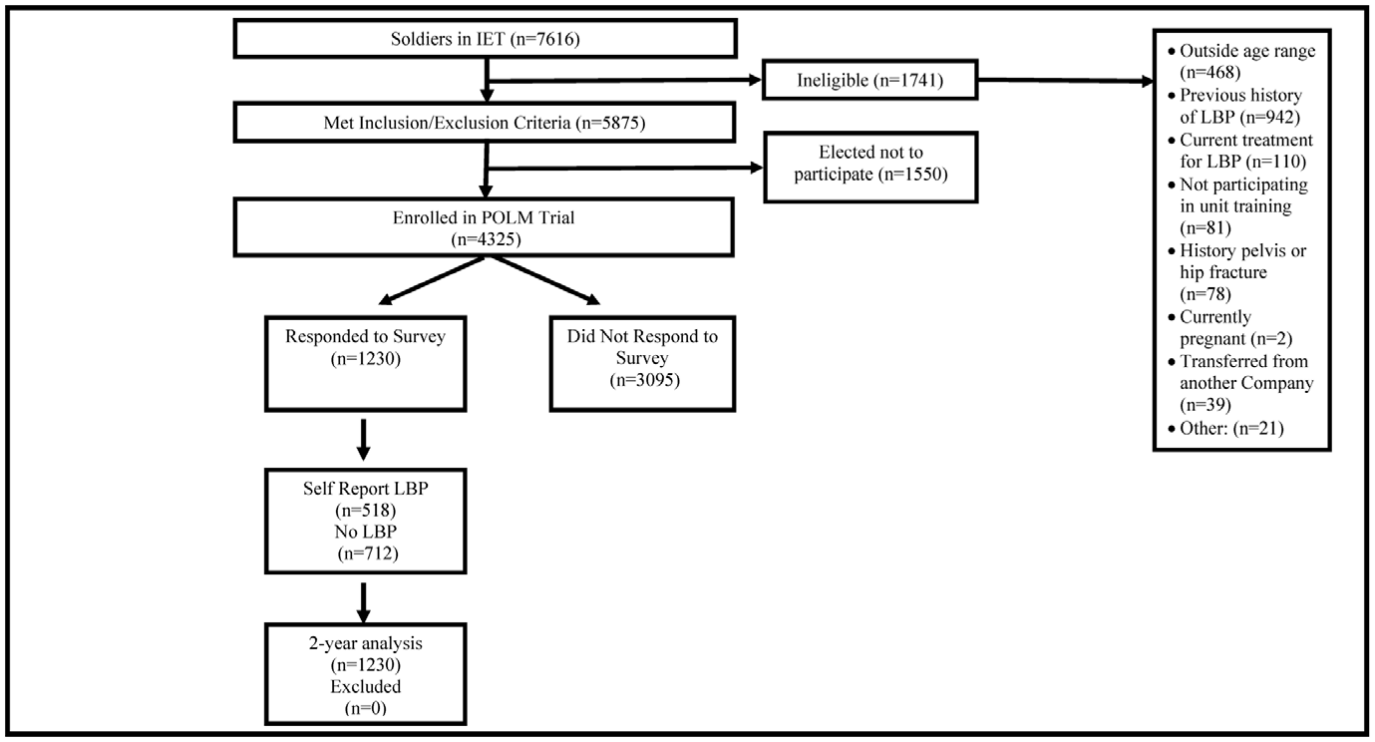
Flow diagram for inception cohort and responders to email surveys. Initial Entry Training (IET), Low Back Pain (LBP), Prevention of Low Back Pain in the Military (POLM), n = total number of soldiers.

Figure 1 describes the recruitment and follow up for the POLM cohort, with 1230/3095 (28.4%) responding to the monthly surveys. Of those responding to the surveys, 518/1230 (42.1%) reported at least one episode of LBP during the 2-year follow up period. Specifically, 420 (48.6%) of 865 Soldiers who responded to the monthly web-based survey had at least one episode of LBP, and corresponding numbers for the phone survey were 129 (24.6%) out of 525 responders. Accompanying descriptive data for the POLM cohort is reported in Tables 1 and 2. Soldiers who responded to the phone or web-based surveys over the 2 years differed from the non-responders in age, race, education level, military status, time in military, negative affect (depressive symptoms and anxiety), back beliefs, mental function, smoking prior to military service, exercising routinely prior to military service, and military fitness scores (Tables 1 and 2). In analyses directly relevant for the purposes of this paper, comparisons of baseline characteristics between those who reported LBP and those who did not report LBP revealed differences in gender, activity duty status, BDI, FPQ, SF-12 PCS, SF-12 MCS, and reporting a previous non LBP related injury (Tables 1 and 2).

**Table 1:** Comparison of baseline innate and psychological characteristics between those who had LBP and those who had no LBP.

Variable	Label	Responders	LBP	No LBP	P-Value*	Non-responders	P-Value#
		n = 1230	n = 518	n = 712		n = 3095	
**Innate Characteristics**							
Age (N = 4319)		22.3±4.5	22.6±4.6	22.1±4.3	0.080	21.9±4.1	**0.001**
Gender	Male	852 (69.6%)	329 (63.9%)	523 (73.7%)	**0.0002**	2230 (72.3%)	0.068
	Female	373 (30.4%)	186 (36.1%)	187 (26.3%)		853 (27.7%)	
Race	Black or Africa	109 (8.9%)	58 (11.2%)	51 (7.2%)	0.059	311 (10.1%)	**0.020**
	Hispanic	100 (8.1%)	37 (7.1%)	63 (8.9%)		326 (10.6%)	
	White or Caucas	927 (75.5%)	381 (73.6%)	546 (76.9%)		2263 (73.3%)	
	Other	92 (7.5%)	42 (8.1%)	50 (7.0%)		187 (6.1%)	
EDUCATION	High school or lower	448 (36.4%)	193 (37.3%)	255 (35.8%)	0.204	1487 (48.1%)	**<0.0001**
	Some college	622 (50.6%)	249 (48.1%)	373 (52.4%)		1376 (44.5%)	
	College or higher	160 (13.0%)	76 (14.7%)	84 (11.8%)		231 (7.5%)	
INCOME	Less than $20,000	609 (49.7%)	249 (48.3%)	360 (50.6%)	0.430	1516 (49.1%)	0.738
	Greater than $20,000	617 (50.3%)	266 (51.7%)	351 (49.4%)		1571 (50.9%)	
Active Duty	Active	568 (46.2%)	263 (50.8%)	305 (42.8%)	**0.012**	1964 (63.5%)	**<0.0001**
	Reserve	660 (53.7%)	255 (49.2%)	405 (56.9%)		1122 (36.3%)	
	Other	2 (0.2%)		2 (0.3%)		6 (0.2%)	
Time In Army	<5 months	684 (55.6%)	287 (55.4%)	397 (55.8%)	0.968	2007 (64.9%)	**<0.0001**
	5 months–1 year	315 (25.6%)	132 (25.5%)	183 (25.7%)		654 (21.2%)	
	More than 1 year	231 (18.8%)	99 (19.1%)	132 (18.5%)		430 (13.9%)	
Company Instructor	Delta	295 (24.0%)	117 (22.6%)	178 (25.0%)	0.482	674 (21.8%)	0.286
	Foxtrot	130 (10.6%)	49 (9.5%)	81 (11.4%)		393 (12.7%)	
	Echo	194 (15.8%)	85 (16.4%)	109 (15.3%)		471 (15.2%)	
	Alpha	185 (15.0%)	79 (15.3%)	106 (14.9%)		442 (14.3%)	
	Charlie	169 (13.7%)	68 (13.1%)	101 (14.2%)		441 (14.2%)	
	Bravo	257 (20.9%)	120 (23.2%)	137 (19.2%)		674 (21.8%)	
Height		68.3±3.9	68.0±3.9	68.4±3.9	0.080	68.3±3.9	0.585
Weight		164.0±27.8	164.0±28.0	164.0±27.7	0.992	165.2±27.6	0.207
BMI		24.7±3.1	24.8±3.1	24.6±3.2	0.184	24.8±3.1	0.194
**Psychological Characteristics**							
BDI Total		6.0±6.1	6.6±5.9	5.5±6.2	**0.002**	6.6±6.7	**0.003**
FPQ Total		18.2±5.7	18.5±5.9	17.9±5.5	**0.040**	18.0±6.0	0.476
BBQ Total		43.9±7.2	43.8±7.2	44.0±7.1	0.610	43.2±7.0	**0.002**
STAI		35.3±9.0	35.9±9.4	34.9±8.6	0.055	36.3±9.2	**0.001**

Responders = those responding to online survey, Non-responders = those not responding to survey, LBP = low back pain,

* = p-values for comparison of those with LBP and those without LBP (responders only),

# = p-values for comparison of those responding to survey and those not responding to survey,

BMI = Body Mass Index, BDI = Beck Depression Inventory, FPQ = Fear of Pain Questionnaire (9 items), BBQ = Back Beliefs Questionnaire, STAI = State Trait Anxiety Inventory (state portion only). Bold font indicates p-value less than 0.05.

**Table 2:** Comparison of baseline health status, activity, and attention effects between those who had LBP and those who had no LBP.

variable	Label	Responders	LBP	No LBP	P-Value*	Non-responders	P-Value#
		n = 1230	n = 518	n = 712		n = 3095	
**Baseline Health Status & Physical Activity**							
PCS Total		53.6±5.0	53.0±5.5	54.0±4.6	**0.001**	53.3±5.2	0.191
MCS Total		49.8±8.0	49.0±8.4	50.4±7.5	**0.002**	48.9±8.8	**0.002**
Smoke Prior to Army	Yes	333 (27.1%)	150 (29.0%)	183 (25.7%)	0.205	1219 (39.4%)	**<0.0001**
	No	897 (72.9%)	368 (71.0%)	529 (74.3%)		1874 (60.6%)	
Exercise Routinely	Yes	670 (54.5%)	278 (53.7%)	392 (55.1%)	0.629	1550 (50.1%)	**0.010**
	No	560 (45.5%)	240 (46.3%)	320 (44.9%)		1542 (49.9%)	
Last APFT Score	Below 150	4 (0.3%)	1 (0.2%)	3 (0.4%)	0.191	20 (0.6%)	**0.021**
	150–200	260 (21.2%)	125 (24.2%)	135 (19.0%)		750 (24.2%)	
	200–250	567 (46.2%)	233 (45.1%)	334 (47.0%)		1461 (47.2%)	
	250–300	369 (30.0%)	149 (28.8%)	220 (30.9%)		808 (26.1%)	
	Above 300	28 (2.3%)	9 (1.7%)	19 (2.7%)		55 (1.8%)	
Profiled	Yes	231 (18.8%)	118 (22.8%)	113 (15.9%)	**0.002**	664 (21.5%)	0.050
	No	999 (81.2%)	400 (77.2%)	599 (84.1%)		2430 (78.5%)	
**Attention/Relational Effect**							
Physical/USI Exam	No	1102 (89.6%)	458 (88.4%)	644 (90.4%)	0.249	2849 (92.1%)	**0.010**
	Yes	128 (10.4%)	60 (11.6%)	68 (9.6%)		246 (7.9%)	
PSEP	No	642 (52.2%)	269 (51.9%)	373 (52.4%)	0.874	1670 (54.0%)	0.294
	Yes	588 (47.8%)	249 (48.1%)	339 (47.6%)		1425 (46.0%)	
Exercise Group	TEP only	334 (27.2%)	148 (28.6%)	186 (26.1%)	0.622	882 (28.5%)	0.747
	TEP+PSEP	277 (22.5%)	119 (23.0%)	158 (22.2%)		675 (21.8%)	
	CSEP	308 (25.0%)	121 (23.4%)	187 (26.3%)		788 (25.5%)	
	CSEP+PSEP	311 (25.3%)	130 (25.1%)	181 (25.4%)		750 (24.2%)	

Responders = those responding to online survey, Non-responders = those not responding to survey, LBP = low back pain,

* = p-values for comparison of those with LBP and those without LBP (responders only),

# = p-values for comparison of those responding to survey and those not responding to survey,

PCS (SF-12) = Physical Component Summary Score from the Short Form Medical Survey (12 items), MCS (SF-12) = Mental Component Summary Score from the Short Form Medical Survey (12 items), APFT = Army Physical Fitness Test, Profiled = injured during training, USI = ultrasound imaging, PSEP = psychosocial education program, TEP = traditional exercise program, CSEP = core stabilization exercise program. Bold font indicates p-value less than 0.05.

### Factors Predictive of First Episode of Self-Reported LBP

**Figure 2:**
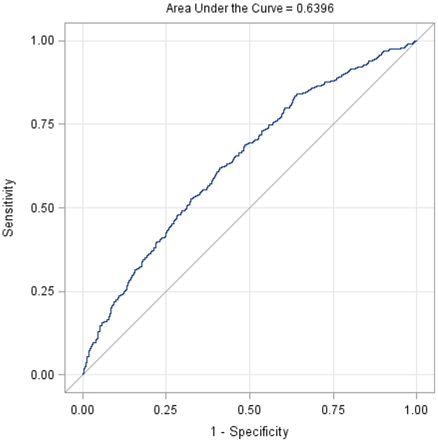
ROC curve for the multivariate logistic regression model predicting initial low back pain occurrence.

Multivariate logistic regression analysis indicated that gender, active duty status, SF-12 PCS, and SF-12 MCS scores were significant predictors of LBP (Table 3). Specifically, protective factors for developing LBP were being male (OR = 0.644, 95% CI = [0.490, 0.846]), and having better SF-12 PCS scores (OR = 0.960 for each additional point, 95% CI = [0.935, 0.987]) and better SF-12 MCS scores (OR = 0.964 for each additional point, 95% CI = [0.943, 0.985]). Increased risk of reporting LBP was noted for Soldiers on active duty (OR = 1.441 times the odds for soldiers on reserve, 95% CI = [1.094, 1.899]). Figure 2 presents the ROC curve for the fitted logistic regression model, which has an area under curve (AUC) of 0.64. Due to correlation between MCS score, STAI and BDI (with correlation coefficients approximately equal 0.60), we compared the above results with those from a reduced model attained through backward variable selection. All four factors remained statistically significant with ORs of 0.626, 0.960, 0.973 and 1.392, respectively. The reduced model also identified BMI as a risk factor (OR = 1.044 for each additional point, 95% CI = [1.005, 1.084]), and it has AUC of 0.61 for its ROC curve. Furthermore, similar sensitivity and specificity were found from 100 repetitions of cross-validations that left out one-third randomly selected samples in the model fitting.

**Table 3:** Logistic regression for baseline prediction of reporting first episode of low back pain.

Factor	Lavel	Odds Ratio Estimates				P-value
		Compare to	Point Estimate	95% Confidence Limits		
Gender	Male	Female	0.644	0.49	0.846	**0.002**
Race	Black or Africa	Other	1.324	0.729	2.405	0.357
	Hispanic	Other	0.761	0.41	1.414	0.389
	White or Caucas	Other	0.949	0.592	1.522	0.829
Education	High school or lower	College or higher	1.013	0.65	1.58	0.954
	Some college	College or higher	0.845	0.573	1.247	0.398
INCOME	Less than $20,000	Greater than $20,000	1.048	0.807	1.361	0.727
Active Duty Status	Active	Reserve	1.441	1.094	1.899	**0.009**
Smoke Prior to entering Army	Yes	No	1.098	0.833	1.448	0.508
Time In Army	<5 months	More than 1 year	0.914	0.654	1.279	0.601
	5 months–1 year	More than 1 year	1.075	0.746	1.551	0.697
Exercise Routinely	Yes	No	1.074	0.834	1.382	0.582
Last APFT Score	Below 150	Above 300	0.357	0.029	4.439	0.423
	150–200	Above 300	1.471	0.603	3.59	0.397
	200–250	Above 300	1.122	0.475	2.654	0.793
	250–300	Above 300	1.163	0.491	2.756	0.731
Previous Injury	Yes	No	1.224	0.891	1.683	0.213
Age			1.012	0.978	1.046	0.498
BMI			1.039	0.998	1.082	0.061
PCS (SF-12)			0.96	0.935	0.987	**0.003**
MCS (SF-12)			0.964	0.943	0.985	**0.001**
FPQ			1.008	0.986	1.031	0.468
STAI			0.982	0.961	1.003	0.089
BDI			1.01	0.982	1.039	0.497
BBQ			1	0.983	1.017	0.966
Exercise and Education Groups	TEP	CSEP	1.372	0.985	1.91	0.061
	TEP+PSEP	CSEP	1.297	0.911	1.845	0.149
	TEP	TEP+PSEP	1.058	0.751	1.489	0.748

LBP = low back pain, BMI = body mass index, BDI = Beck Depression Inventory, FPQ = Fear of Pain Questionnaire (9 items), BBQ = Back Beliefs Questionnaire, STAI = State Trait Anxiety Inventory (state portion only), PCS (SF-12) = Physical Component Summary Score from the Short Form Medical Survey (12 items), MCS (SF-12) = Mental Component Summary Score from the Short Form Medical Survey (12 items), TEP = traditional exercise program, PSEP = psychosocial education program, CSEP = core stabilization exercise program. Bold font indicates p-value less than 0.05.

Cox regression revealed that the time to first episode of LBP was significantly shorter for female, active duty, profiled soldiers and increasing BMI, with corresponding hazard ratios of 1.497 (95% CI = [1.238, −1.809]), 1.366 (95% CI = [1.120, 1.665]), 1.271 (95% CI = [1.026, 1.573]) and 1.031 (95% CI = [1.002, 1.060]), respectively.

### Factors Predictive of LBP Severity – Pain Intensity

Descriptive statistics for pain intensity reported during first LBP episode were 2.0 (sd = 1.9) for the current pain intensity rating, 3.2 (sd = 2.5) for the highest pain intensity rating in past 24 hours, and 0.8 (sd = 1.4) for the lowest pain intensity rating in the past 24 hours. Multivariate linear regression analysis identified baseline predictors of current (R^2^ = 7.4%), highest (R^2^ = 9.9%), and lowest (R^2^ = 9.7%) NRS pain intensity ratings during the first episode of LBP. Only education level and last physical fitness score were predictors of all NRS pain intensity ratings. Duration of service and better SF-12 MCS score were also predictors for highest NRS pain intensity ratings. Specifically, highest pain intensity ratings for soldiers with high school or lower education levels were 1.121 higher (95% CI = [0.239, 2.003] on average than ratings for soldiers with college degrees, 0.948 higher (95% CI = [0.186, 1.710]) for soldiers with 5 months to 1 year of service compared to those with more than 1 year of service, and 5.560 higher (95% CI = [0.117, 11.003]) on average than ratings for soldiers with physical fitness scores below 150 when compared to those above 300. SF-12 MCS scores were protective of highest pain intensity ratings, with a decrease of 0.043 for each additional point (95% CI = [−0.086, 0.001]).

### Factors Predictive of LBP Severity – Disability

Descriptive statistics for disability reported during first LBP episode were 9.8 (sd = 11.7) for ODQ score and 1.3 (sd = 4.5) for number of days with limited work duties in the past 30 days. Multivariate linear regression analysis revealed gender, smoking status, and previous injury status as baseline predictors of ODQ score (R^2^ = 11.4%) and days of limited work duty (R^2^ = 6.2%). Specifically, men on average scored 3.118 points lower on the ODQ than women (95% CI = [−5.646, −0.590]) while Soldiers who smoked prior to entering the Army scored on average 2.671 higher than those who did not (95% CI = [0.0270, 5.315]). Soldiers who had a previous non LBP related injury scored on average 3.394 higher on the ODQ than soldiers who had not (95% CI = [0.505, 6.283]). The number of days of limited duty, on average, was 1.6 less for Soldiers in the TEP only group than for soldiers receiving combined CSEP+PSEP group (95% CI = [−2.858, −0.342]).

### Factors Predictive of LBP Severity – Psychological Distress

Descriptive statistics for psychological distress reported during first LBP episode were 9.7 (sd = 6.1) for FABQ-PA scores, 10.6 (sd = 9.1) for FABQ-W scores, and 5.5 (sd = 9.5) for CAT scores. Multivariate linear regression analysis indicated that gender, smoking status, and SF-12 PCS scores were predictive of FABQ-PA scores (R^2^ = 14.2%) while BMI, SF-12 PCS, and BBQ scores were predictive of FABQ-W scores (R^2^ = 10.6%). For the FABQ-PA men on average scored 1.639 lower than women (95% CI = [−2.952, −0.326]) and Soldiers who smoked scored on average 1.700 higher than those who did not smoke (95% CI = [0.332, 3.068]). FABQ-PA scores decreased on average by 0.183 points for each additional point of SF-12 PCS (95% CI = [−0.303, −0.0634]). FABQ-W scores decreased on average by 0.590 for each unit increase in BMI (95% CI = [−0.911, −0.269]) and by 0.224 points for each additional point of SF-12 PCS (95% CI = [−0.406, −0.0417]). FABQ-W scores also decreased on average by 0.133 for each additional point of BBQ (95% CI = [−0.260, −0.0056]).

Multivariate linear regression analysis indicated that exercising routinely before entering the military, SF-12 PCS, and the BBQ were predictive of CAT scores (R^2^ = 8.6%) during first episode of LBP. Specifically, we estimated that CAT scores are 2.380 points higher on average for soldiers who exercised routinely (95% CI = [0.347, 4.413]), while CAT scores decreased by 0.239 for each additional point of SF-12 PCS (95% CI = [−0.433, −0.0450], p = 0.016). CAT scores also decreased by 0.143 for each additional point of BBQ score (95% CI = [−0.280, −0.0058], p = 0.040).

## Discussion

Rigorous studies reporting predictors for the development of LBP are rarely reported in the literature because of the difficulty of assembling pain free cohorts and following them until LBP occurs. Strengths of the current study were the recruitment of a large inception cohort of Soldiers without previous history of LBP, consideration of a range of potentially relevant baseline predictors, and collection of follow up data over a 2 year period. Results from this study suggest that active duty (i.e. not reserve or national guard) status increased risk of LBP occurrence, while better mental and physical health scores and being male were protective. Being female and active duty status were also predictive of shorter time to first episode of LBP, as well as experiencing a previous non LBP related injury and having higher BMI. In studies that used a similar prospective design we found agreement with better physical health [38], [39], [40] as being protective for the development of LBP. Other studies reported risk factors that were not predictive of occurrence in this cohort including age [41], [40], body weight [39], [40], smoking status [42], and psychological or psychosocial factors [43], [42], [44]. Our military cohort was of a relatively homogenous age range, with low baseline levels of psychological and psychosocial factors. Obviously cohort differences could account for the discrepancies in identified predictors of LBP occurrence, as there is limited consistency among these studies.

A novel aspect of the current study was that we were also able to identify predictors of severity for the first LBP episode by collecting data on pain intensity, disability, and psychological distress. Collectively our results indicated that lower educational and fitness levels were predictive of higher pain intensity scores. Smoking and history of previous non LBP related injury were predictive of having more disability, while being male was predictive of less disability. Only the type of exercise program was predictive of number of days of missed duty. Lower education and fitness levels consistently predicted higher psychological distress, while better physical health scores and beliefs about back pain were predictive of lower psychological distress. There were two counterintuitive findings for psychological distress noted. Higher BMI was predictive of lower work fear-avoidance beliefs and exercising routinely was predictive of higher pain catastrophizing. Overall these results considering severity of first LBP episode may provide important new data on prevention strategies, as previous studies in this area have focused exclusively on incidence or occurrence of LBP [39], [43], [41], [42], [38], [44], [40]. Data from the current study extends previous work by highlighting factors that may predict severity of first LBP episode. These factors may be especially important for future prevention studies because severity appears to be a key factor in the decision to seek healthcare for LBP [45], [46], [47]. Comparison of these factors to others reported in the literature was not possible because incidence studies have not typically included severity measures. Therefore these predictors of severity should be considered as preliminary with future research necessary to replicate these findings.

Although we are reporting baseline predictors of the development and severity of LBP, several caveats should be considered when interpreting these results. First, there were very few consistent predictors identified in this study. Many of the predictors appeared to be specific to the outcome measure. Second, we identified baseline predictors that were statistically significant, but the magnitude of these predictors was often low. For example, the increased risk of being on active duty was associated with an OR of 1.44 (lower bound of 95% CI = 1.09), while the protective factor of being male was associated with an OR of 0.64 (upper bound of 95% CI = 0.85). Third, we included all participants in our prediction models instead of including only those in a control condition as is more commonly done in prognostic studies. This decision was made because in this setting all Soldiers were undergoing training which included required exercise so there was no option for a “true” control condition of no exercise. Last, not many of the identified predictive factors were modifiable in nature. This was especially true for the factors predicting the occurrence of LBP, with more opportunity for modifiable factors noticed for the severity outcomes. Data from this cohort provide further indication of the multi-factorial nature of the development of LBP as inconsistency in predictors seems to be the norm in the literature, [39], [43], [41], [42], [38], [44], [40]. In the event consistent factors are identified they often lack the magnitude to be considered as “definitive” predictors for the development of LBP.

Effective strategies for preventing LBP remain elusive. Back schools, lumbar supports, and ergonomic interventions have limited evidence for prevention [14], [13], [15] Education for primary prevention of LBP has received mixed support in trials; with support for psychosocial education [17], but not for biomedical or biomechanical based education programs [14]. Collectively the results from the current study and others investigating predictors of LBP [39], [43], [41], [42], [38], [44], [40] provide guidance for future primary prevention intervention strategies. It is clear from the assembled data that single modality approaches are not likely to be effective in preventing LBP, echoing expert and consensus opinion in this area [6], [48]. A recommendation from the current study is that LBP prevention strategies will likely have to be contextual in nature, such that effective LBP prevention strategies for a military population may differ from one used for hospital nurses. For example, results from this military cohort suggest prevention of occurrence of LBP may be futile based on the lack of modifiable predictors, but there were some modifiable factors identified in a study of nurses [44]. Based on our data preventative interventions tailored at decreasing the severity of LBP or visits for health care seem feasible for the military, especially those that utilize general exercise approaches to target improving fitness levels and educational programs to improve back beliefs [17], [23].

There are, of course, limitations to consider when interpreting results from this study. The primary limitation is the low follow up rate to our web based survey system (28.4%) which did not allow us to follow the entire cohort. We have identified differences in responders in a preliminary analysis at 1 year follow up [49] and these differences were confirmed in this paper with 2 year follow up. However we do not have specific reasons for the lack of response. It could be due to the wide geographic area of deployment for these Soldiers including areas that would not have ready email access. We did consider using estimation or imputation methods to account for these missing data. However our confidence in the validity of these techniques to provide additional information was low due to the follow up rate (not enough complete data to allow for estimation or imputation) and the aforementioned baseline differences between responders (data used for estimating or imputing) and non-responders. Therefore, a decision was made to report only the completed data and note the limitations of doing so, rather than taking on additional limitations inherent in estimating missing data in a situation like this.

The result of the low follow up rate highlights the difficulty of collecting complete data on inception cohorts for the development of LBP. Critical interpretation of our results hinges on the acknowledgment that responder bias may have had a profound impact on these analyses. There are numerous baseline differences in responders and non-responders and this is a concern. Many of these statistical differences were small in magnitude and likely a function of the high statistical power that accompanies a large sample. For example the age difference between responders and non-responders was 0.4 years corresponding to an effect size of 0.09 (Cohen's D). Such a small difference in age between responders and non responders is not likely to reflect responder bias for age, and there were similarly small differences for many of the other variables (Tables 1 and 2). Another issue to consider when determining the impact of the low follow up rate is that our methodology differed from other survey studies in that we had access to the entire sample at baseline (Figure 1). We were then able to determine differences between survey responders and non responders. This type of comparison is not an option in the more commonly incorporated design of surveying a larger group with the researchers only able to analyze data from responders. In the current study we had the advantage of being able to identify characteristics of non-responders so that the reader can make his/her own conclusions about the impact of responder bias. Unfortunately the definite quantitative impact of this responder bias for this study is impossible to estimate. In practical terms it means that these data can only be generalized to individuals that respond to survey requests, which makes it quite consistent with other studies in the literature that using this methodology.

Another consequence of this low follow up rate is that our estimates of LBP occurrence should not be mistaken for a true incidence estimate for this population. It is quite likely that the rate of LBP (42.1%) in this cohort is an overestimate of the actual incidence rate. Another potential limitation is that these analyses focused only on the first time episode of LBP. We did not consider recurrence in these analyses, but acknowledge that LBP is often a recurrent problem [50]. Finally another limitation to consider is the validity of self-report of LBP has been questioned for military populations as discrepancies were identified in indication of ever having LBP and scores on validated questionnaires (like the ODQ) [51]. This concern is mitigated somewhat in the current study because we didn't rely solely on one aspect of self-report, and instead included multiple validated questionnaires.

In conclusion, this military inception cohort provided information on baseline factors that were predictive of occurrence of LBP and severity. Our results confirm the multi-factorial nature of LBP as predictors of LBP occurrence and severity were neither consistent nor definitive. Future attempts at developing preventative strategies should consider these results and focus on multi-modal approaches that target modifiable risk factors specific to the population of interest.

## References

[1] Mantyselka P, Kumpusalo E, Ahonen R, Kumpusalo A, Kauhanen J (2001). Pain as a reason to visit the doctor: a study in Finnish primary health care.. Pain.

[2] Walker BF, Muller R, Grant WD (2004). Low back pain in Australian adults: prevalence and associated disability.. J Manipulative Physiol Ther.

[3] Stewart WF, Ricci JA, Chee E, Morganstein D, Lipton R (2003). Lost productive time and cost due to common pain conditions in the US workforce.. JAMA.

[4] Cohen SP, Brown C, Kurihara C, Plunkett A, Nguyen C (2010). Diagnoses and factors associated with medical evacuation and return to duty for service members participating in Operation Iraqi Freedom or Operation Enduring Freedom: a prospective cohort study.. Lancet.

[5] Lincoln AE, Smith GS, Amoroso PJ, Bell NS (2002). The natural history and risk factors of musculoskeletal conditions resulting in disability among US Army personnel.. Work.

[6] Burton AK, Balague F, Cardon G, Eriksen HR, Henrotin Y (2005). How to prevent low back pain.. Best Pract Res Clin Rheumatol.

[7] Cohen SP, Nguyen C, Kapoor SG, Anderson-Barnes VC, Foster L (2009). Back pain during war: an analysis of factors affecting outcome.. Arch Intern Med.

[8] Frank JW, Brooker AS, DeMaio SE, Kerr MS, Maetzel A (1996). Disability resulting from occupational low back pain. Part II: What do we know about secondary prevention? A review of the scientific evidence on prevention after disability begins.. Spine.

[9] Nicholas MK, Linton SJ, Watson PJ, Main CJ (2011). Early Identification and Management of Psychological Risk Factors (“Yellow Flags”) in Patients With Low Back Pain: A Reappraisal.. Phys Ther.

[10] Foster NE, Hill JC, Hay EM (2011). Subgrouping patients with low back pain in primary care: are we getting any better at it?. Man Ther.

[11] Fritz JM, Cleland JA, Childs JD (2007). Subgrouping patients with low back pain: evolution of a classification approach to physical therapy.. J Orthop Sports Phys Ther.

[12] Frank JW, Kerr MS, Brooker AS, DeMaio SE, Maetzel A (1996). Disability resulting from occupational low back pain. Part I: What do we know about primary prevention? A review of the scientific evidence on prevention before disability begins.. Spine.

[13] Linton SJ, van Tulder MW (2001). Preventive interventions for back and neck pain problems: what is the evidence?. Spine.

[14] Burton AK, Balague F, Cardon G, Eriksen HR, Henrotin Y (2006). Chapter 2. European guidelines for prevention in low back pain : November 2004.. Eur Spine J.

[15] Bigos SJ, Holland J, Holland C, Webster JS, Battie M (2009). High-quality controlled trials on preventing episodes of back problems: systematic literature review in working-age adults.. Spine J.

[16] George SZ, Childs JD, Teyhen DS, Wu SS, Wright AC (2007). Rationale, design, and protocol for the prevention of low back pain in the military (POLM) trial (NCT00373009).. BMC Musculoskelet Disord.

[17] George SZ, Childs JD, Teyhen DS, Wu SS, Wright AC (2011). Briefy psychosocial education, not core stabilization, reduced incidence of low back pain: results from the Prevention of Low Back Pain in the Military (POLM) cluster randomized trial.. BMC Med.

[18] Childs JD, Teyhen DS, Benedict TM, Morris JB, Fortenberry AD (2009). Effects of sit-up training versus core stabilization exercises on sit-up performance.. Med Sci Sports Exerc.

[19] Childs JD, Teyhen DS, Casey PR, Coy-Singh KA, Feldtmann AW (2010). Effects of traditional sit-up training versus core stabilization exercises on short-term musculoskeletal injuries in US Army soldiers: a cluster randomized trial.. Phys Ther.

[20] Burton AK, Waddell G, Tillotson KM, Summerton N (1999). Information and advice to patients with back pain can have a positive effect. A randomized controlled trial of a novel educational booklet in primary care.. Spine.

[21] Coudeyre E, Tubach F, Rannou F, Baron G, Coriat F (2007). Effect of a simple information booklet on pain persistence after an acute episode of low back pain: a non-randomized trial in a primary care setting.. PLoS ONE.

[22] George SZ, Fritz JM, Bialosky JE, Donald DA (2003). The effect of a fear-avoidance-based physical therapy intervention for patients with acute low back pain: results of a randomized clinical trial.. Spine.

[23] George SZ, Teyhen DS, Wu SS, Wright AC, Dugan JL (2009). Psychosocial education improves low back pain beliefs: results from a cluster randomized clinical trial (NCT00373009) in a primary prevention setting.. Eur Spine J.

[24] Ware J, Kosinski M, Keller SD (1996). A 12-Item Short-Form Health Survey: construction of scales and preliminary tests of reliability and validity.. Med Care.

[25] Symonds TL, Burton AK, Tillotson KM, Main CJ (1996). Do attitudes and beliefs influence work loss due to low back trouble?. Occup Med (Lond).

[26] Spielberger CD, Gorsuch RL, Lushene RE, Vagg PR, Jacobs GA (1983). Manual for the state and trait anxiety inventory (form Y).

[27] Whisman MA, Perez JE, Ramel W (2000). Factor structure of the Beck Depression Inventory-Second Edition (BDI-II) in a student sample.. J Clin Psychol.

[28] Schotte CK, Maes M, Cluydts R, De Doncker D, Cosyns P (1997). Construct validity of the Beck Depression Inventory in a depressive population.. J Affect Disord.

[29] Chibnall JT, Tait RC (1994). The short form of the Beck Depression Inventory: validity issues with chronic pain patients.. Clin J Pain.

[30] McNeil DW, Rainwater AJ (1998). Development of the Fear of Pain Questionnaire–III.. J Behav Med.

[31] Albaret MC, Munoz Sastre MT, Cottencin A, Mullet E (2004). The Fear of Pain questionnaire: factor structure in samples of young, middle-aged and elderly European people.. Eur J Pain.

[32] Osman A, Breitenstein JL, Barrios FX, Gutierrez PM, Kopper BA (2002). The Fear of Pain Questionnaire-III: further reliability and validity with nonclinical samples.. J Behav Med.

[33] Jensen MP, Turner JA, Romano JM (1994). What is the maximum number of levels needed in pain intensity measurement?. Pain.

[34] Fairbank JC, Pynsent PB (2000). The Oswestry Disability Index.. Spine.

[35] Roland M, Fairbank J (2000). The Roland-Morris Disability Questionnaire and the Oswestry Disability Questionnaire.. Spine.

[36] Waddell G, Newton M, Henderson I, Somerville D, Main CJ (1993). A Fear-Avoidance Beliefs Questionnaire (FABQ) and the role of fear-avoidance beliefs in chronic low back pain and disability.. Pain.

[37] Sullivan MJL, Bishop SR, Pivik J (1995). The Pain Catastrophizing Scale: development and validation.. Psychological Assessment.

[38] Stevenson JM, Weber CL, Smith JT, Dumas GA, Albert WJ (2001). A longitudinal study of the development of low back pain in an industrial population.. Spine.

[39] Croft PR, Papageorgiou AC, Thomas E, Macfarlane GJ, Silman AJ (1999). Short-term physical risk factors for new episodes of low back pain. Prospective evidence from the South Manchester Back Pain Study.. Spine.

[40] Elders LA, Burdorf A (2004). Prevalence, incidence, and recurrence of low back pain in scaffolders during a 3-year follow-up study.. Spine.

[41] Park H, Sprince NL, Whitten PS, Burmeister LF, Zwerling C (2001). Risk factors for back pain among male farmers: analysis of Iowa Farm Family Health and Hazard Surveillance Study.. Am J Ind Med.

[42] Power C, Frank J, Hertzman C, Schierhout G, Li L (2001). Predictors of low back pain onset in a prospective British study.. Am J Public Health.

[43] Papageorgiou AC, Macfarlane GJ, Thomas E, Croft PR, Jayson MI (1997). Psychosocial factors in the workplace–do they predict new episodes of low back pain? Evidence from the South Manchester Back Pain Study.. Spine.

[44] Yip VY (2004). New low back pain in nurses: work activities, work stress and sedentary lifestyle.. J Adv Nurs.

[45] Cote P, Cassidy JD, Carroll L (2001). The treatment of neck and low back pain: who seeks care? who goes where?. Med Care.

[46] Mortimer M, Ahlberg G (2003). To seek or not to seek? Care-seeking behaviour among people with low-back pain.. Scand J Public Health.

[47] IJzelenberg W, Burdorf A (2004). Patterns of care for low back pain in a working population.. Spine.

[48] Guzman J, Hayden J, Furlan AD, Cassidy JD, Loisel P (2007). Key factors in back disability prevention: a consensus panel on their impact and modifiability.. Spine (Phila Pa 1976).

[49] Childs JD, Teyhen DS, Van Wyngaarden JJ, Dougherty BF, Ladislas BJ (2011). Predictors of web-based follow-up response in the Prevention of Low Back Pain the Military Trial (POLM).. BMC Musculoskelet Disord.

[50] Carey TS, Garrett JM, Jackman A, Hadler N (1999). Recurrence and care seeking after acute back pain: results of a long-term follow-up study. North Carolina Back Pain Project.. Med Care.

[51] Carragee EJ, Cohen SP (2009). Lifetime asymptomatic for back pain: the validity of self-report measures in soldiers.. Spine (Phila Pa 1976).

